# The contrasting roles of PPARδ and PPARγ in regulating the metabolic switch between oxidation and storage of fats in white adipose tissue

**DOI:** 10.1186/gb-2011-12-8-r75

**Published:** 2011-08-11

**Authors:** Lee D Roberts, Andrew J Murray, David Menassa, Tom Ashmore, Andrew W Nicholls, Julian L Griffin

**Affiliations:** 1 Department of Biochemistry University of Cambridge, Tennis Court Road, Cambridge CB2 1QW, UK; 2 The Cambridge Systems Biology Centre, University of Cambridge, Tennis Court Road, Cambridge CB2 1QR, UK; 3 Department of Physiology, Development and Neuroscience University of Cambridge, Downing Street, Cambridge CB2 3EG, UK; 4 GlaxoSmithKline, Investigative Preclinical Toxicology, Park Road, Ware, SG12 0DP, UK; 5 MRC Human Nutrition Research, Elsie Widdowson Laboratory, Fulbourn Road, Cambridge, CB1 9NL, UK; 6 The MRC Centre for Obesity and Related Disorders (CORD), Institute of Metabolic Sciences, University of Cambridge, Addenbrooke's Hospital, Cambridge, CB2 0QQ, UK

## Abstract

**Background:**

The nuclear receptors peroxisome proliferator-activated receptor γ (PPARγ) and peroxisome proliferator-activated receptor δ (PPARδ) play central roles in regulating metabolism in adipose tissue, as well as being targets for the treatment of insulin resistance. While the role of PPARγ in regulating insulin sensitivity has been well defined, research into PPARδ has been limited until recently due to a scarcity of selective PPARδ agonists.

**Results:**

The metabolic effects of PPARγ and PPARδ activation have been examined *in vivo *in white adipose tissue from *ob*/*ob *mice and *in vitro *in cultured 3T3-L1 adipocytes using ^1^H nuclear magnetic resonance spectroscopy and mass spectrometry metabolomics to understand the receptors' contrasting roles. These steady state measurements were supplemented with ^13^C-stable isotope substrate labeling to assess fluxes, in addition to respirometry and transcriptomic microarray analysis. The metabolic effects of the receptors were readily distinguished, with PPARγ activation characterized by increased fat storage, synthesis and elongation, while PPARδ activation caused increased fatty acid β-oxidation, tricarboxylic acid cycle rate and oxidation of extracellular branch chain amino acids. Stimulated glycolysis and increased fatty acid desaturation were common pathways for the agonists.

**Conclusions:**

PPARγ and PPARδ restore insulin sensitivity through varying mechanisms. PPARδ activation increases total oxidative metabolism in white adipose tissue, a tissue not traditionally thought of as oxidative. However, the increased metabolism of branch chain amino acids may provide a mechanism for muscle atrophy, which has been linked to activation of this nuclear receptor. PPARδ has a role as an anti-obesity target and as an anti-diabetic, and hence may target both the cause and consequences of dyslipidemia.

## Background

The World Health Organization estimates over 180 million people worldwide suffer from type 2 diabetes mellitus (T2DM). The incidence of obesity, a major risk factor for the development of T2DM, is also increasing globally. While a number of anti-diabetic treatments have been produced, they rarely address the related obese state and consequently fail to confront this underlying risk factor. Therefore, it becomes imperative that new treatment approaches with both anti-diabetic and anti-obesity properties are found.

The peroxisome proliferator-activated receptors (PPARs) are ligand activated transcription factors, belonging to the nuclear receptor superfamily, that control the expression of genes involved in organogenesis, inflammation, cell differentiation, proliferation, and lipid and carbohydrate metabolism [1]. Activation of the PPARs by their selective ligands results in heterodimerization of the receptor with the 9-*cis*-retinoic acid receptor. The PPARs can then bind to specific sequences in their target genes known as peroxisome proliferator response elements [2].

There are three distinct PPAR subtypes, PPARα, PPARγ and PPARδ, with each demonstrating a particular tissue distribution and ligand specificity [3]. PPARα is primarily expressed in heart, liver, macrophages and intestines, and is activated by polyunsaturated fatty acids and leukotriene B4 [4]. PPARγ is principally expressed in adipocytes but is also found in a range of tissues, including the placenta. The receptor has a key role in adipocyte differentiation and lipid storage; it is activated by polyunsaturated fatty acids and 15d-prostaglandin J2 [5]. PPARδ is expressed almost ubiquitously, though some tissues express higher concentrations of the mRNA, including the brain, skin, liver, skeletal muscle and adipose tissue [6,7]. In recent studies, the vitamin A metabolite retinoic acid has been identified as a physiological ligand for the PPARδ nuclear receptor, acting to control cell survival [8].

The PPARs have already yielded viable targets for the treatment of T2DM and dyslipidemia; thiazolidinediones, PPARγ agonists, are currently used in the clinic for the treatment of T2DM; and fibrates, PPARα agonists, are routinely used to treat dyslipidemia. Treatment with thiazolodinediones results in the recruitment of new metabolically active adipocytes, causing an increase in lipid storage capacity and normalization of adipocytokine levels [9].

A pharmacological agonist for PPARδ is yet to make it into the clinic and the receptor remains to be fully functionally defined. However, the development of a number of high affinity synthetic ligands for PPARδ has shown the receptor holds considerable promise for the treatment of T2DM, the metabolic syndrome, dyslipidemia and obesity. Insulin-resistant obese rhesus monkeys treated with the selective PPARδ agonist GW501516 demonstrated significant increases in high-density lipoprotein cholesterol with concomitant decreases in triacylglycerols (TAGs) and low-density lipoprotein cholesterol [10]. PPARδ activation has also shown efficacy in reducing adiposity by decreasing intracellular triglyceride accumulation in mouse brown adipose tissue and liver [11].

Investigation into the function of PPARδ in white adipose tissue has demonstrated that the receptor has an important role in the regulation of metabolism. Tissue-specific over-expression of PPARδ in the white adipose tissue of transgenic mice resulted in a decrease in body weight, adipocyte triglyceride accumulation, circulating free fatty acids and circulating triglyceride [11]. The same transgenic mice were also protected against weight gain, adipocyte hypertrophy, hypertriglyceridemia, and steatosis. PPARδ activation also leads to elevated expression of *uncoupling protein-1 *in white adipose tissue [11].

In order to contrast the roles of PPARγ and PPARδ in regulating metabolism in white adipose tissue, we have performed a metabolomics study using both *in vivo *analysis in the *ob*/*ob *mouse and *in vitro *analysis using the murine 3T3-L1 adipocyte cell line. The *ob*/*ob *mouse was used to investigate the influence of PPAR activation on adipose tissue metabolism in a model of insulin resistance and obesity. The *ob*/*ob *mouse model is robust, well characterized and used extensively to study T2DM and its therapies; however, it is worthy of note that it is a monogenic paradigm of leptin deletion, whereas T2DM is a polygenic disorder.

A synthetic, high affinity pharmacological agonist, GW610742, was used to activate PPARδ in both the mice and the adipocyte cell line (GW610742 EC50 for murine PPARδ is 28 nM compared to 8,900 nM for PPARα and > 10,000 nM for PPARγ) [12] and contrasted with a well defined PPARγ agonist (GW347845). Steady state concentrations were assessed *in vivo *and *in vitro *using a combination of mass spectrometry (MS) and ^1^H nuclear magnetic resonance (^1^H NMR) spectroscopy in conjunction with multivariate statistics to probe the metabolic phenotypes resulting from activation of the two nuclear receptors. To unambiguously define the mechanisms by which PPARδ and PPARγ alter the metabolism of adipose tissue, this was further characterized by ^13^C-stable isotope substrate labeling studies using 1-^13^C glucose and U-^13^C palmitate, respirometric analysis using a Clark-type oxygen electrode and transcriptomic microarray analysis.

It was found that PPARδ activation was characterized not only by increased fatty acid oxidative metabolism as previously observed but also by increased glucose and amino acid oxidation. In contrast, activation of PPARγ was associated with fatty acid synthesis and sequestration of fats. This implicates PPARδ as a control for global oxidative energy metabolism and suggests a mechanism by which activation of the nuclear receptor, in part, brings about its anti-diabetic and anti-obesity properties by simultaneously reducing the quantity of triglycerides and glucose in white adipose tissue and systemic metabolism as a whole. However, this metabolic systems biology approach also suggests that increased demand for branched chain amino acids (BCAAs) in adipose tissue may explain why the wider metabolic effects of PPARδ activation may cause muscle atrophy.

## Results

### Metabolomic analysis of adipose tissue from *ob*/*ob *mice treated with the PPAR agonists

A combination of gas chromatography (GC)-MS and direct infusion (DI)-MS combined with multivariate pattern recognition was used to profile metabolism within the white adipose tissue of *ob*/*ob *mice treated with either GW610742 (a selective PPARδ agonist), GW347845 (a selective PPARγ agonist) or a vehicle control. These analytical approaches provided coverage of total fatty acids and intact lipids and free fatty acids, respectively. The various spectra and chromatograms were interrogated using multivariate statistics comparing the dosed groups with the vehicle control.

**Figure 1:**
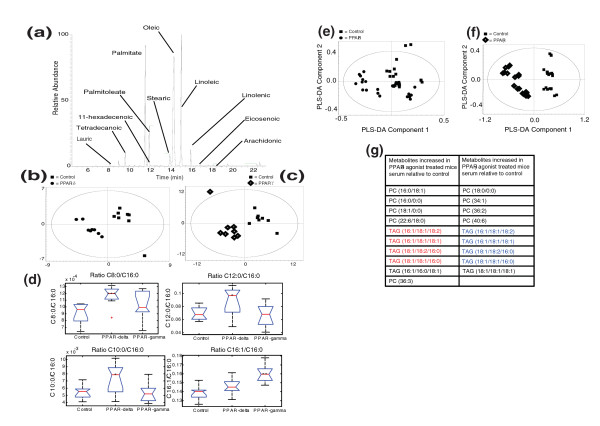
**Metabolomic investigation of PPARδ and PPARγ activation in white adipose tissue from *ob*/*ob *mice**. **(a) **Chromatogram of GC-MS analysis of the total fatty acid content of white adipose tissue from an *ob*/*ob *mouse treated with the PPARδ agonist. Key metabolites are labeled. **(b) **Partial least squares-discriminant analysis (PLS-DA) of the GC-MS chromatograms from white adipose tissue from control animals (filled squares; *n *= 8) or those treated with a PPARδ (filled circles; *n *= 8) (R^2^(X) = 32%, Q^2 ^= 69%). **(c) **PLS-DA of the GC-MS chromatograms from white adipose tissue from control animals (filled squares; *n *= 8) or those treated with the PPARγ agonist (diamonds; *n *= 8) (R^2^(X) = 32%, Q^2 ^= 74%). **(d) **Box whisker plots of key metabolic changes in total fatty acids in white adipose tissue following treatment with either the PPARδ agonist (*n *= 8) or PPARγ agonist (*n *= 8). Significant differences were measured by ANOVA followed by a Tukey post-hoc test. *P < 0.05; **P < 0.01; ***P < 0.005. **(e) **Plot of PLS-DA scores showing the clustering of DI-MS negative ionization mode mass spectra run in triplicate from the organic phase of white adipose extracts from *ob*/*ob *mice treated with a PPARδ agonist compared with control animals: PPARδ agonist-treated (filled circles; *n *= 8), control (filled squares; *n *= 8) (R^2^(X) = 72%, Q^2 ^= 58%). **(f) **Plot of PLS-DA scores showing the clustering of DI-MS positive ionization mode mass spectra run in triplicate from the organic phase of white adipose extracts from *ob*/*ob *mice treated with a PPARγ agonist compared with control animals: PPARγ agonist-treated (diamonds; *n *= 8), control (filled squares; *n *= 8) (R^2 ^= 89%, Q^2 ^= 95%). **(g) **Key metabolic changes detected by liquid chromatography-MS in blood serum from animals treated with either a PPARδ agonist (*n *= 8) or PPARγ agonist (*n *= 8) compared with wild-type controls (*n *= 8). The metabolite changes demonstrate a restructuring of specific lipid species, particularly phosphatidylcholines (PC) and triacylglycerols (TAG), within the circulating lipid pool of PPARδ and PPARγ agonist-treated mice. The TAG species increased in the PPARδ agonist-treated mice marked in red are decreased in the PPARγ agonist-treated mice marked in blue.

Both PPARδ and PPARγ agonists induced large changes in the total fatty acid profile of white adipose tissue as measured by GC-MS of the fatty acid methyl esters and subsequent multivariate analysis (Figure 1a-c). Treatment with the PPARδ agonist induced decreases in the medium-chain fatty acids, while the concentration of the shorter chain fatty acids increased (Figure 1d). This was contrasted by the effect of PPARγ where the most profound change was an increase in activity of Δ-9 desaturase, increasing the concentrations of desaturated fatty acids, as well as an increase in the long chain fatty acid arachidate C20:0.

Analysis of the DI-MS negative mode ionization data of the organic phase was used to analyze changes in free fatty acids and a number of classes of intact lipids, with this approach distinguishing both adipose tissue from *ob*/*ob *mice treated with either of the agonists (Figure 1e, f). Interrogating the loadings plots of the multivariate models, both agonists stimulated increases in the constituents of the ω-6 fatty acid pathway, demonstrating that both agonists stimulate the activity of desaturases. However, the most profound difference between the two agonists was characterized by decreased concentrations of the long chain saturated fatty acids (C19:0, C20:0, C21:0 and C22:0) following PPARδ agonist treatment, while free palmitic acid, stearic acid and its desaturated forms (C18:1 and C18:2) increased in compensation.

Given the important role adipose tissue plays in modulating the lipid composition of blood serum, liquid chromatography (LC)-MS was used to profile intact lipids in blood serum (Figure 1g). While both agonists induced changes in polar lipids, the most dramatic contrast was apparent in changes in the TAG content. The PPARδ agonist induced increases in the concentration of a number of circulating TAG species containing C16:0, C18:0 and C18:1 fatty acids in blood serum, while the same TAG species were decreased following PPARγ agonist treatment (Figure 1g). Thus, stimulation of PPARδ increased the mobilization of TAGs, and PPARγ stimulation increased the sequestration of TAGs.

These metabolic changes demonstrate that while both agonists induced the activity of desaturases, the PPARδ agonist was characterized by a reduction in fatty acid chain length consistent with increased β-oxidation. However, because both agonists influence metabolism in a range of organs, this study was complemented with an analysis of 3T3-L1 adipocytes to examine adipocyte metabolism in isolation.

### Metabolomic analysis of 3T3-L1 adipocytes treated with the PPAR agonists

**Figure 2:**
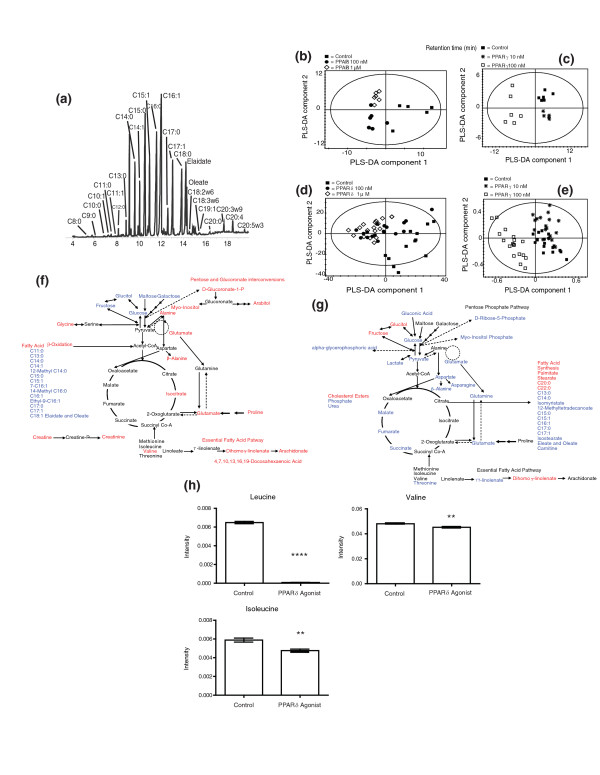
**Metabolomic investigation of PPARδ and PPARγ activation in 3T3-L1 adipocytes**. **(a) **Chromatogram of GC-MS analysis of the total fatty acid content of 3T3-L1 adipocytes treated with the PPARδ agonist. Key metabolites are labeled. **(b) **Plot of partial least squares-discriminant analysis (PLS-DA) scores showing the clustering of GC-MS chromatograms from the lipid fraction of 3T3-L1 adipocytes treated with 100 nM and 1 μM PPARδ agonist GW610742 compared with the control group: 1 μM PPARδ agonist dose (diamonds; *n *= 6), 100 nM PPARδ agonist dose (filled circles; *n *= 6), control (filled squares; *n *= 6) (R^2^(X) = 77%, Q^2 ^= 75%). **(c) **Plot of PLS-DA scores showing the clustering of GC-MS chromatograms from the organic fraction of 3T3-L1 adipocytes treated with 10 nM PPARγ agonist GW347845 and 100 nM PPARγ agonist GW347845 compared with the control group: 10 nM PPARγ agonist dose (asterisks; *n *= 6), 100 nM PPARγ agonist dose (squares; *n *= 6), control (filled squares; *n *= 6) (R^2^(X) = 87%, Q^2 ^= 90%). **(d) **Plot of PLS-DA scores showing the clustering of DI-MS negative mode ionization chromatograms from the organic fraction of 3T3-L1 adipocytes treated with 100 nM and 1 μM PPARδ agonist GW610742 compared with the control group: 1 μM PPARδ agonist dose (diamonds; *n *= 6), 100 nM PPARδ agonist dose (filled circles; *n *= 6), control (filled squares; *n *= 6) (R^2^(X) = 70%, Q^2 ^= 85%). **(e) **Plot of PLS-DA scores showing the clustering of DI-MS negative mode ionization chromatograms from the organic fraction of 3T3-L1 adipocytes treated with 10 nM PPARγ agonist GW347845 and 100 nM PPARγ agonist GW347845 compared with the control group: 10 nM PPARγ agonist dose (asterisks; *n *= 6), 100 nM PPARγ agonist dose (squares; *n *= 6), control (filled squares; *n *= 6) (R^2^(X) = 86%, Q^2 ^= 88%). **(f) **Key steady state metabolic changes detected in 3T3-L1 adipocytes following treatment with the PPARδ agonist GW610742 using a combination of ^1^H NMR spectroscopy and GC-MS. Metabolites increased in concentration are labeled in red, and metabolites decreased in concentration are labeled in blue. **(g) **Key steady state metabolic changes detected in 3T3-L1 adipocytes following treatment with the PPARγ agonist GW347845 using a combination of ^1^H NMR spectroscopy and GC-MS. Metabolites increased in concentration are labeled in red, and metabolites decreased in concentration are labeled in blue. (**h) **Changes in BCAAs in the culture media of PPARδ agonist-treated 3T3-L1 cells **P < 0.005, ****P < 0.0001. Error bars represent standard errors of the mean.

To profile total fatty acid changes, GC-MS of fatty acid methyl esters was again applied in conjunction with multivariate statistics (Figure 2a). The loadings plots of the partial least squares-discriminant analysis (PLS-DA) models of the data were again used to determine the key metabolic changes in total fatty acid profiles induced by the two agonists (Figure 2b, c). Similar to the changes detected in adipose tissue, both agonists affected the ω-6 fatty acid pathway, with the PPARδ agonist increasing the concentrations of a number of the later pathway intermediates, while the PPARγ agonist decreased γ-linolenate, and increased dihomo-γ-linolenate. PPARδ activation also stimulated an increase in the end products of the ω-3 fatty acid pathway (C20:5, C22:5 and C22:6).

However, the major difference between the two agonists was a general decrease in the concentration of fatty acids observed in PPARδ agonist-treated cells) while PPARγ stimulation induced a relative change in overall chain length characterized by decreases in the concentrations of the medium chain fatty acids (C13:0, C14:0, C15:0, C16:1, C17:0, C17:1, C18:1) and a concomitant increase in the steady state concentrations of the long chain fatty acids (C20:0 and C22:0). These changes in total fatty acids were also represented in the free fatty acid profile measured by DI-MS and modeled by multivariate analysis (Figure 2d, e).

Changes in the composition of complex lipids was observed in the PPARδ activated 3T3-L1 adipocytes. An increase in the concentration of a number of glycerophosphocholine and phosphatidylcholine (PCs) species was ascertained and this was accompanied by a decrease in the concentration of specific TAGs (Table 1). Unlike the shift from TAGs to phospholipids induced by PPARδ, the activation of PPARγ produced a more complex remodeling of TAGs with an increase in longer chain and desaturated fatty acids, which dominated the resultant PLS-DA model (Table 1). In addition, a range of PCs, glycerophosphocholines, glycerophosphoethanolamines and glycerophosphoinsoitols decreased in concentration while the concentration of several cholesterol esters increased (data not shown).

**Table 1:** Lipid species altered in concentration in 3T3-L1 adipocytes treated with either the PPARδ agonist GW610742 or the PPARγ agonist GW347845

PPARδ		PPARγ	
**Increased**	**Decreased**	**Increased**	**Decreased**

PC 32:0 (16:0/16:0)	TAG 52:1	TAG 48:0	TAG 44:2
PC 34:0	TAG 52:5	TAG 50:1	TAG 44:1 (15:0/15:0/14:1)
PC 34:1	TAG 52:6	TAG 52:4	TAG 44:1 (15:1/14:0/15:0)
PC 35:5	TAG 53:2	TAG 54:6	TAG 45:2
PC 36:1	TAG (18:3/17:0/19:0)	TAG 54:5	TAG 46:2
PC 36:2	TAG (18:1/17:1/19:1)	TAG 54:4	TAG 47:2
PC 36:3	TAG (20:1/17:1/17:1)		TAG 47:3
	TAG (20:1/15:0/19:2)		TAG 48:3
	TAG (20:1/15:1/19:1)		TAG 48:2
			TAG 49:3
			TAG 50:3

Species were detected using LC-MS. Lipids identified in the VIP/coefficient plots as significantly contributing to separation in the principal components analysis (PCA) and PLS-DA models built for the LC-MS analysis of the organic metabolite fraction (*P *< 0.05 for significant contribution to the first component of the PLS-DA plot). The control group (*n *= 6) was compared with the PPARδ agonist-treated group (*n *= 6) or PPARγ agonist-treated group (*n *= 6). All triacylglycerols (TAGs) were observed as ammonium adducts. Where stated, exact composition was confirmed by tandem mass spectrometry (MS/MS) and phosphocholines (PCs) were identified by monitoring for the loss of the choline head group during MS/MS.

A combination of both NMR spectroscopy and GC-MS analysis of aqueous metabolites readily distinguished the action of the two PPAR receptors. PPARδ activation increased the concentration of the peroxisomal oxidation product adipic acid, while PPARγ stimulation decreased the concentration of carnitine, the main transporter of fatty acids across the mitochondrion. The PPARδ agonist also decreased the concentration of glucose and other carbohydrates in adipose cells, as well as increased the concentration of citrate and glutamate (the latter in fast exchange with 2-oxogluturate). While PPARγ stimulation also decreased the concentration of glucose, it also decreased the concentrations of the later tricarboxylic acid (TCA) cycle metabolites. Changes in the steady state concentrations of specific metabolites and corresponding metabolic pathways in 3T3-L1 adipocytes treated with either the PPARδ or PPARγ agonist are summarized in Figure 2f, g.

To assess how metabolism in the 3T3-L1 cells influenced their environments, metabolite changes in the media were investigated using a combination of GC-MS, probing fatty acid export from the cells, and ^1^H NMR spectroscopy, determining changes in aqueous phase metabolites. While the PPARδ agonist did not affect fatty acid export compared with cells treated with the vehicle control, PPARγ reduced the export of fatty acids, particularly of saturated fatty acids (palmitate, *P *= 0.01, 23% reduction; stearate, *P *= 0.04, 19% reduction). However, PPARδ activation markedly reduced the concentrations of amino acids in the PPARδ cell culture media compared with both the control group and cells treated with the agonist, in particular the BCAAs leucine (*P *< 0.0001), isoleucine (*P *= 0.002) and valine (*P *= 0.005) (Figure 2h). Concomitantly, the steady state intracellular concentration of valine was increased in PPARδ agonist-treated cells (*P *< 0.05)

### ^13^C-labelled substrate studies

In order to identify the metabolic mechanisms associated with PPARδ and PPARγ activation in white adipose tissue and 3T3-L1 adipocytes, the ^13^C-labeled substrates 1-^13^Cglucose and U-^13^C-palmitate were used to monitor flux through glycolytic and fatty acid oxidative pathways.

**Figure 3:**
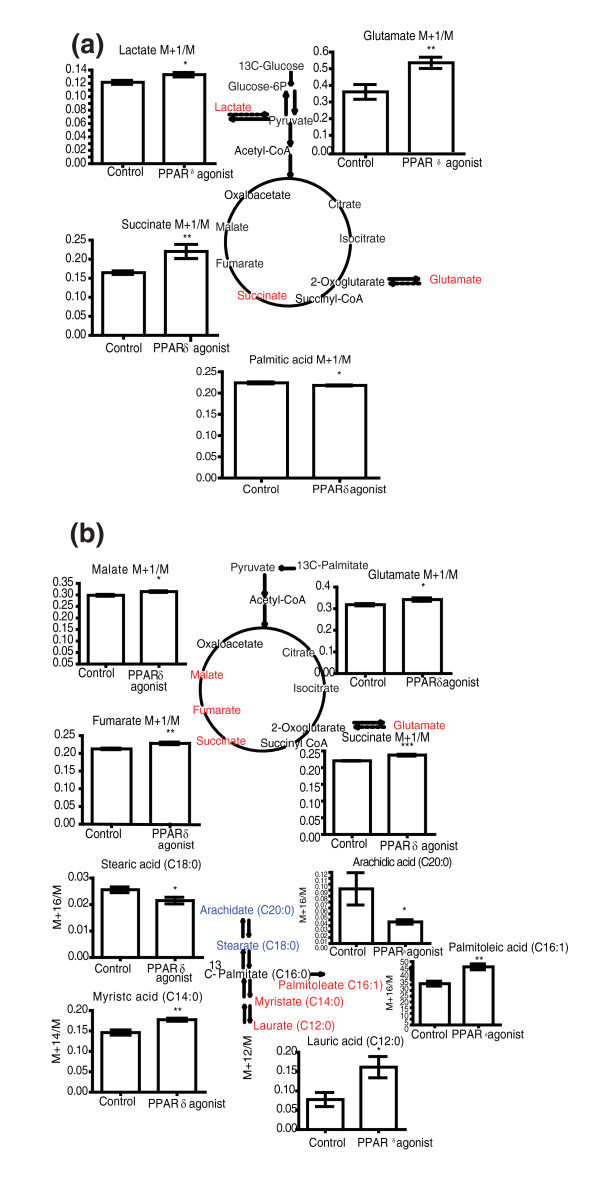
**Stable isotope flux analysis of PPARδ agonist-treated 3T3-L1 adipocytes**. **(a) **Graphs showing the M+1/M isotope ratio ^13^C enrichment of lactate, glutamate and succinate analyzed by GC-MS of the aqueous fraction and M+1/M isotope ratio ^13^C enrichment of palmitic acid analyzed by GC-MS of the organic fraction from control (*n *= 6) and PPARδ agonist-treated (*n *= 6) 3T3-L1 cells incubated with 1-^13^C glucose. *P < 0.05, **P < 0.01. The metabolites have been mapped to the glycolysis and TCA cycle metabolic pathways. Red indicates a metabolite increased in ^13^C enrichment by PPARδ activation. **(b) **Graphs showing the M+1/M isotope ratio ^13^C enrichment of malate, glutamate, fumarate and succinate analyzed by GC-MS of the aqueous fraction and enrichment of arachidic acid, stearic acid, palmitoleic acid, myristic acid and lauric acid analyzed by GC-MS of the organic fraction from control (*n *= 6) and PPARδ agonist-treated (*n *= 6) 3T3-L1 cells incubated with U-^13^C palmitate. *P < 0.05, **P < 0.01,***P < 0.005. Red indicates a metabolite increased, and blue indicates a metabolite decreased in ^13^C enrichment by PPARδ activation. Parent ions were used to calculate ion ratio. Error bars represent standard errors of the mean.

The use of 1-^13^C glucose and GC-MS readily distinguished the two agonists. Examination of the aqueous phase by GC-MS revealed that lactate, glutamate (readily labeled from the TCA cycle from labeled 2-oxoglutarate) and succinate from PPARδ agonist-treated cells were enriched with ^13^C when compared to control (Figure 3a). In contrast, the PPARγ agonist caused a reduction in labeling of lactate, succinate and glutamate compared to the vehicle-treated cells (Additional file 1). The PPARδ agonist also decreased labeling of the medium chain fatty acid palmitate from 1-^13^C glucose, while the PPARγ agonist increased the labeling of the long chain fatty acid arachidate. In addition, ^13^C NMR spectroscopy of the organic fraction of control and PPARδ agonist-treated adipocytes incubated in media containing 1-^13^C glucose showed that glycerol and esterified glycerol from PPARδ agonist-treated cells had reduced enrichment compared with the control group (Additional file 2).

Similarly, the labeled substrate U-^13^C palmitate readily distinguished the two agonists. Assessment of the aqueous phase by GC-MS indicated that several TCA cycle intermediates from PPARδ agonist-treated adipocytes were enriched compared to control cells (Figure 3b). Investigation of the organic phase by GC-MS demonstrated that the fatty acids downstream of palmitic acid in the β-oxidation pathway showed greater ^13^C enrichment in PPARδ agonist-treated cells; as did the Δ-9 desaturation product of palmitic acid. Simultaneously, the enrichment of fatty acids upstream of palmitic acid in the fatty acid synthesis pathway was reduced in PPARδ agonist-treated cells (Figure 3b).

GC-MS analysis of the aqueous phase of cells incubated in U-^13^C palmitate indicated that the early TCA cycle intermediates exhibited decreased ^13^C enrichment in PPARγ agonist-treated adipocytes when compared to control adipocytes (Additional file 1). Assessment of the organic phase by GC-MS indicated that the ^13^C enrichment of the long chain fatty acid arachidate was increased in the PPARγ agonist-treated cells when compared to control (Additional file 1). Concurrently, the ^13^C enrichment of the shorter chain fatty acid myristate was decreased.

### Respirometric analysis

**Figure 4:**
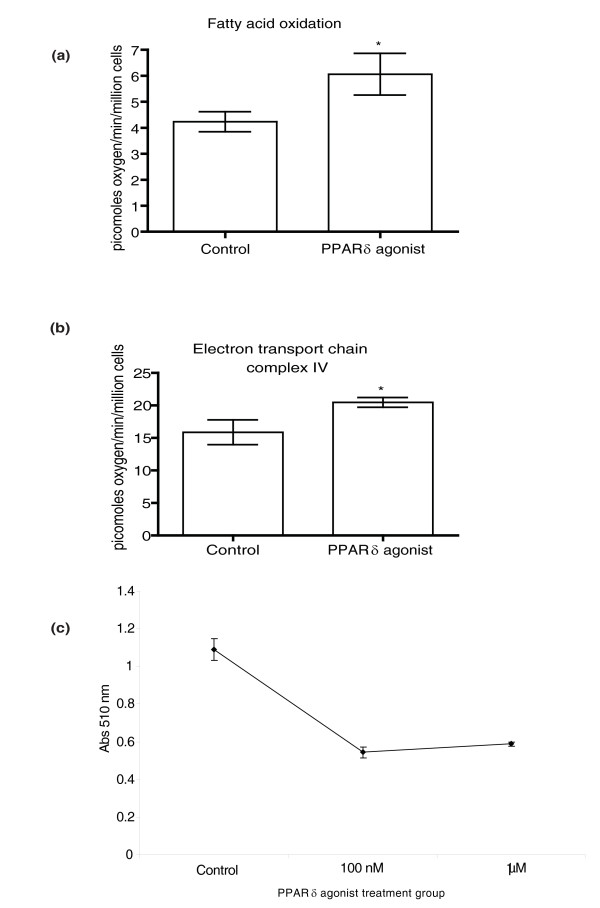
**Respirometric analysis of PPARδ agonist-treated 3T3-L1 adipocytes**. **(a) **Graph showing the respiratory rates of *in situ *permeabilized control (*n *= 3) and PPARδ agonist-treated (*n *= 3) 3T3-L1 cells performing β-oxidation using palmitoyl-carnitine measured using a Clark-type oxygen electrode. *P = 0.05. **(b) **Graph showing the respiratory rates of the electron transport chain complex IV of *in situ *permeabilized control (*n *= 3) and PPARδ agonist-treated (*n *= 3) 3T3-L1 cells measured using a Clark-type oxygen electrode. *P < 0.05. **(c) **Spectrophotometric measurement at 510 nm of Oil Red O eluted from stained 3T3-L1 cells treated with DMSO control (*n *= 3) or 100 nM (*n *= 3) or 1 μM (*n *= 3) of the PPARδ agonist GW610742. Error bars represent standard errors of the mean.

To further characterize the PPARδ induced upregulation of oxidative pathways in adipocytes, the oxygen consumption of PPARδ agonist-treated and control 3T3-L1 cells was measured both when using fatty acid as substrate and during isolated electron transport chain complex IV oxidation using *in situ *studies in a Clarke type oxygen electrode. Both complex IV and fatty acid oxidation were significantly increased in the adipocytes exposed to the PPARδ agonist when compared to control adipocytes (Figure 4a, b). This was accompanied by a profound decrease in TAGs as measured by Oil Red O staining of neutral lipids (Figure 4c).

### Microarray transcriptomic analysis

**Figure 5:**
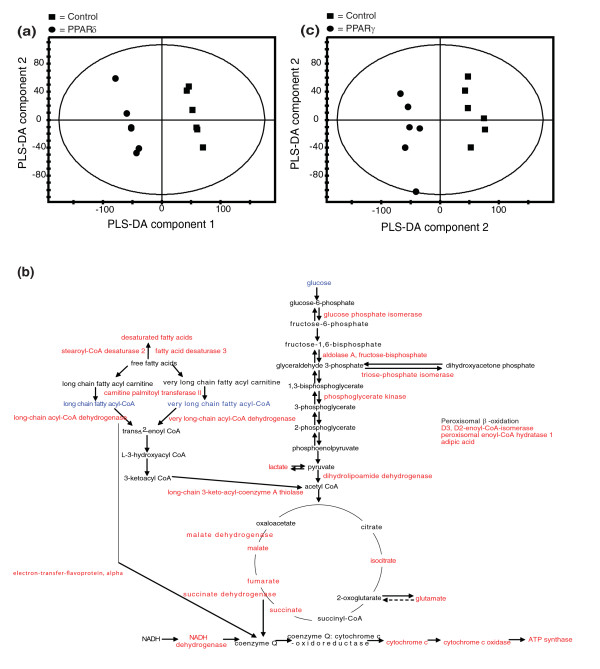
**Transcriptomic analysis of PPARδ and PPARγ activation in 3T3-L1 adipocytes**. **(a) **Plot of PLS-DA scores showing the clustering of gene transcription in control and PPARδ agonist-treated 3T3-L1 adipocytes as measured with microarray analysis: PPARδ agonist-treated (filled circles; *n *= 6), control (filled squares; *n *= 6) (R^2^(X) = 35%, Q^2 ^= 90%). **(b) **Diagram showing the effect of PPARδ activation on the integration of the energy metabolism pathways of 3T3-L1 adipocytes based on the combination of results from the metabolomic, transcriptomic and stable isotope labeling studies. Red indicates an increase in concentration or expression in cells treated with the PPARδ selective agonist GW610742. Blue indicates a decrease in concentration in cells treated with the PPARδ selective agonist GW610742. **(c) **Plot of PLS-DA scores showing the clustering of gene transcription in control and PPARγ agonist-treated 3T3-L1 adipocytes as measured with microarray analysis: PPARγ agonist-treated (filled circles; *n *= 6), control (filled squaresl *n *= 6) (R^2^(X) = 42%, Q^2 ^= 84%).

The combination of steady state metabolomic changes in adipose tissue and adipocytes and isotope labeling studies indicated a profound upregulation of glucose and fatty acid oxidation following PPARδ activation. To investigate these changes in more detail, we moved focus to the transcriptome using microarray analysis of PPARδ activation in adipocytes. Of the 45,281 probes utilized, 13,718 were expressed above the background defined by the negative control probe. From these, 2,349 were determined to be differentially expressed with a 95% confidence level between PPARδ agonist-treated and control 3T3-L1 adipocytes. In addition to the univariate analysis, multivariate models were also built using the total normalized data (Figure 5a). The 6% of transcripts most responsible for separation in the multivariate models were then examined (3% with the highest positive contribution to principal component 1 and 3% with the highest negative contribution to principal component 1 in PPARδ agonist-treated cells as identified in the multivariate models). The multivariate analysis indicated that the mRNA of genes involved in a number of key metabolic pathways was altered following PPARδ activation. The Reactome Skypainter tool was then utilized to determine which pathways and reactions were statistically overrepresented by the 3% most increased and 3% most decreased transcripts in PPARδ agonist-treated cells identified in the multivariate models (Table 2; Figure 5b) [13].

**Table 2:** The pathways statistically significant in the 3% most increased transcripts in PPARδ agonist-treated cells identified in the multivariate models

P-value	Pathway	Transcripts increased in PPARδ agonist-treated cells mapping to the pathway
6.3e-08	Glucose regulation of insulin secretion	*Cycs*, *Etfa*, *Mdh2*, ***Aldoa***, *Dld*, *Ndufb10*, *Ndufb9*, *Atp5a1*, *Ndufb5*, ***Gpi1***, *Tpi1*, *Pgk1*, *mt-Co2*, *Sdhb*, *Sdhd*, *mt-Atp6*, *Cox7b*, *Ndufb2*
1.3e-06	Integration of energy metabolism	*Cycs*, *Etfa*, *Mdh2*, *Aldoa*, *Dld*, *Ndufb10*, *Ndufb9*, *Atp5a1*, *Cpt2*, *Ndufb5*, ***Gpi1***, *Tpi1*, *Pgk1*, *mt-Co2*, *Sdhb*, *Sdhd*, *mt-Atp6*, *Cox7b*, *Ndufb2*
1.3e-06	Diabetes pathways	*Hspa8*, *Cycs*, *Wdr89*, *Etfa*, *Mdh2*, ***Aldoa***, *Dld*, *Ndufb10*, *Myo5a*, *Ndufb9*, *Atp5a1*, *Rps21*, *Rps3a*, *Sec11c*, *Ndufb5*, ***Gpi1***, *Tpi1*, *Pgk1*, *Sdhb*, *mt-Co2*, *Dnajb9*, *Sdhd*, *mt-Atp6*, *Cox7b*, *2900062L11Rik*, *Ndufb2*
8.5e-06	Electron transport chain	*Cycs*, *Ndufb5*, *Etfa*, *mt-Co2*, *Ndufb10*, *Sdhb*, *Sdhd*, *Ndufb9*, *Cox7b*, *Ndufb2*
7.1e-04	Citric acid cycle (TCA cycle)	*Dld*, *Sdhb*, *Sdhd*, *Mdh2*
1.4e-03	Mitochondrial fatty acid β-oxidation of saturated and unsaturated fatty acids	*Hadhb*, *Acadl*, *Acadvl*
1.6e-03	Glycolysis	*Aldoa*, *Tpi1*, *Pgk1*, *Gpi1*
4.5e-03	Metabolism of lipids and lipoproteins	*Agpat3*, *Hadhb*, *Ppp1cc*, ***Slc27a1***, *Lass2*, ***Angptl4***, *Cpt2*, *Akr1b3*, ***Abcd3***, *Acadl*, *Sgpl1*, ***Acaa2***, *Acadvl*, *Mod1*, *Hmgcs2*, *Adfp*
7.8e-03	Formation of acetoacetic acid in synthesis of ketone bodies	*Hmgcs2*, ***Acaa2***

Transcripts in bold were increased in both PPARδ and PPARγ agonist-treated cells.

The expression of genes encoding proteins involved in the mitochondrial β-oxidation pathway and the peroxisomal fatty acid β-oxidation pathway was increased in PPARδ agonist-treated cells. Alongside these changes were an increase in the transcription of genes involved in both mitochondrial and peroxisomal biogenesis and maintenance. The transcription of several genes whose products play a role in the glycolytic metabolic pathway and the TCA cycle was also upregulated in PPARδ agonist-treated cells. In addition, there was a detected increase in the concentrations of expressed mRNA for components of the electron transport chain, and genes involved in fatty acid desaturation (Table 2; Additional file 3).

The results from the steady state metabolomic experiments in adipose tissue and adipocytes and the isotope labeling studies suggest PPARγ activation has a pronounced effect on glucose utilization and fatty acid synthesis and metabolism in adipocytes. Transcriptional changes were investigated by DNA microarrays to further define the changes associated with PPARγ activation in adipocytes. Of the 45,281 probes utilized, 13,755 were expressed above the background defined by the negative control probe. From these, 3,282 were determined to be differentially expressed with a 95% confidence limit. Multivariate models were then built using the total normalized data (Figure 5c). The 6% of transcripts most responsible for separation in the multivariate models (that is, those contributing most to the total variance of the multivariate model) were then examined using a combination of multivariate analysis and the Reactome Skypainter tool as described above [13]. The pathways and reactions that were statistically overrepresented by the 3% most increased and 3% most decreased transcripts in PPARγ agonist-treated cells identified in the multivariate models are shown in Table 3, Figure 5c, and Additional file 4.

**Table 3:** The pathways statistically significant in the 3% most increased transcripts in PPARγ agonist-treated cells identified in the multivariate models

P-value	Pathway	Transcripts increased in PPARγ agonist-treated cells mapping to the pathway
6.4e-05	Glycolysis	***Aldoa***, *Pgam1*, *Pfkl*, *Gapdh*, ***Gpi1***
1.1e-03	Gluconeogenesis	***Aldoa***, *Slc25a11*, *Pgam1*, *Gapdh*, ***Gpi1***
1.2e-03	Ca^2+ ^signaling via IP3 binding to the IP3 receptor, opening the endoplasmic reticulum Ca^2+ ^channel	*Itpr2*, *Itpr1*
1.8e-03	Phospholipase C-mediated signaling events	*Prkaca*, *Itpr2*, *Itpr1*, *Adcy6*, *Pde1b*
2.5e-03	Hormone-sensitive lipase-mediated triacylglycerol hydrolysis	*Prkaca*, *Abhd5*, *Ppp1ca*
2.6e-03	Metabolism of lipids and lipoproteins	*Prkaca*, *Abhd5*, *Abcd3*, *Ppp1ca*, *Ncor1*, *Chd9*, ***Slc27a1***, *Hsd17b4*, ***Acaa2***, *Sin3b*, *Scd2*, *Hmgcl*, *Fads3*, *Csnk1g2*, ***Angptl4***
3.2e-03	Regulation of insulin secretion	*Prkaca*, *Dlst*, *Ndufb6*, *Itpr1*, ***Gpi1***, ***Aldoa***, *Ndufs6*, *Pgam1*, *Itpr2*, *Pfkl*, *Gapdh*
5.8e-03	Formation of acetoacetic acid in the synthesis of ketone bodies	*Hmgcl*, ***Acaa2***
8.2e-03	Additional metabolism of carbohydrates	***Aldoa***, *Slc25a11*, *Pgam1*, *Pfkl*, *Gapdh*, ***Gpi1***, *G6pdx*
1.1e-02	Protein kinase A-mediated events	*Prkaca*, *Pde1b*
1.1e-02	Regulation of lipid metabolism by peroxisome proliferator-activated receptor alpha	*Sin3b*, *Scd2*, *Fads3*, *Ncor1*, *Chd9*, ***Slc27a1***, ***Angptl4***
1.7e-02	Integration of energy metabolism	*Prkaca*, *Dlst*, *Ndufb6*, ***Gpi1***, ***Aldoa***, *Ndufs6*, *Pgam1*, *Pfkl*, *Adcy6*, *Gapdh*
2.0e-02	β-Oxidation of very long chain fatty acids	***Abcd3***, *Hsd17b4*
0.1e-02	Peroxisomal lipid metabolism	***Abcd3***, ***Slc27a1***, *Hsd17b4*

Transcripts in bold were increased in both PPARδ and PPARγ agonist-treated cells.

The expression of genes encoding proteins involved in the glycolytic metabolic pathway were upregulated in PPARγ agonist-treated cells. In addition, the expression of the gene encoding the TCA cycle enzyme isocitrate dehydrogenase was identified as decreased, suggesting that citrate was being channeled to fatty acid synthesis rather than being metabolized by the TCA cycle. PPARγ activation was also discerned to significantly affect the transcription of genes responsible for the remodeling and metabolism of lipids. Genes for fatty acid desaturases (*Scd2 *and *Fads3*) were increased in expression following PPARγ activation. The transcripts of a number of genes that favor conditions of fatty acid synthesis were also increased in concentration in the adipocytes following treatment with the PPARγ agonist. Concomitantly, the expression of genes encoding enzymes that catalyze the hydrolysis of medium and long chain acyl-CoAs to free fatty acids and coenzyme A (CoA) was upregulated in the treated adipocytes (*Acot7 *and *Nudt19*). In addition, the transcription of an insulin responsive fatty acid transporter gene (*Slc27a1*) responsible for the import of long chain fatty acids into adipose tissue undergoing high levels of TAG synthesis was increased.

Several genes involved in the restructuring and remodeling of complex lipids were also affected by PPARγ activation. There was an increase in transcription of genes encoding enzymes responsible for conversion of lysophospholipids to phospholipids, favoring polyunsaturated fatty acyl-CoAs as acyl donors (lysophosphatidylcholine acyltransferase 3 acyltransferase). In addition, mRNA transcripts of genes encoding products that regulate lipolysis, alongside other metabolic processes, including gluconeogenesis, were increased in the PPARγ agonist-treated cells (*platelet activating factor acetylhydrolase 2 lipase*, *angiopoietin-related protein 4 *and *nuclear receptor corepressor 1*). Additionally, transcription of the PPAR transcriptional coactivator gene *chromodomain helicase DNA binding protein 9 *was upregulated in the PPARγ agonist-treated adipocytes.

Several transcripts were increased in both PPARδ and PPARγ agonist-treated cells, principally involved with glycolysis and lipid metabolism. However, PPARδ activation was unique in its effect on the citric acid cycle, the electron transport chain and fatty acid β-oxidation (Tables 2 and 3).

## Discussion

A comprehensive array of analytical techniques was used in a metabolomic investigation to study the metabolic changes occurring in white adipose tissue from *ob*/*ob *mice and 3T3-L1 adipocytes following either PPARδ or PPARγ activation, to understand the role of these nuclear hormone receptors in treating T2DM and obesity. Our metabolomic analysis demonstrates the large differences between the action of the two receptors, with PPARδ associated with a profound increase in oxidation of glucose, fats and amino acids, and PPARγ associated with the sequestration and restructuring of lipids within adipose tissue. This was confirmed by not only observing changes in steady state metabolite concentrations but also using stable isotope techniques to probe flux, oxygen consumption measurements and monitoring transcriptional changes, with agreement across these different tiers of biological organization.

PPARδ activation is known to increase the oxidation of fatty acids [11,14] and this was confirmed by both the relative increase in short chain fatty acids compared with long chain fatty acids in both adipose tissue and 3T3-L1 cells as well as respiration rate measurements and the increased concentration of adipic acid, the primary end product of peroxisomal β-oxidation [15], in cells. Transcriptomic analysis also indicated an increase in β-oxidation, with increases in the transcription of a panel of genes involved in fatty acid mitochondrial and peroxisomal β-oxidation (Table 2; *Cpt2*, *Acadvl*, *Acadl*, *Hadhb*, *Acaa2*, *Abcd3*, *Ech1*, *Peci*). The oxidation of fats was directly followed by monitoring the metabolism of U-^13^C palmitate, with increased labeling of shorter chain fatty acids following the stimulation of PPARδ. PPARδ stimulation also increased the TCA cycle rate, as indicated by following the labeling patterns induced by 1-^13^C glucose, the increased labeling of TCA cycle intermediates during metabolism of U-^13^C palmitate, and the increased steady state concentrations of TCA cycle intermediates in 3T3-L1 cells. Transcriptomic analysis substantiated this finding, with the expression of genes with roles in glycolysis and the TCA cycle significantly increased upon PPARδ activation (Table 2; *Tpi1*, *Gpi1*, *Aldoa*, *Pgk1*, *Mdh2*, *Sdhb*, *Sdhd*, *Dld*).

Wang and colleagues [11] discussed the upregulation of fatty acid oxidation in brown adipose tissue following the over expression of PPARδ in terms of the expression of a range of enzymes involved in uncoupling (uncoupling protein 1 and 3), fatty acid oxidation (acyl-CoA oxidase, muscle carnitine palmitoyltransferase-1, long chain acyl dehydrogenase, very long chain acyl dehydrogenase) as well as morphological changes within the tissue. However, at the transcriptional level there was only an increase in hormone-sensitive lipase in white adipose tissue, despite there being reductions in adipose mass and increased oxygen consumption in the equivalent cell line. While the authors still infer increased oxidation of fatty acids in white adipose tissue, our metabolomic analysis demonstrates this *in vivo *in terms of the changes of metabolites involved in fatty acid oxidation, demonstrating the pharmacological differences between different PPAR agonists, as well as the fact that steady state changes in metabolite concentrations can be as sensitive as transcriptional changes induced by a perturbation.

One possible driving force for this increased oxidation of both glucose and fats, and increased flux through β-oxidation and the TCA cycle, is the increased expression of the components of the electron transport chain (Table 2; Ndufb5, Ndufb10, Ndufb9, Ndufb2, Cys, Etfa, mt-Co2, Sdhb, Sdhd, Cox7b, mt-Atp6). This finding in adipocytes is concordant with previous studies performed in skeletal muscle that demonstrated that PPARδ activation increases the expression of several electron transport chain proteins, including cytochrome c and cytochrome oxidase [16]. Furthermore, respirometry studies confirmed that independently of fatty acid oxidation the oxidative rate of electron transport chain complex IV was increased upon PPARδ activation. This effect was independently observed by the increase in the creatine concentration within 3T3-L1 adipocytes treated with the PPARδ agonist, demonstrating an alteration to the high energy phosphate buffering capacity of the cells. These findings are consistent with previously reported observations in skeletal muscle. A decrease in the intramyocellular lipid-to-total creatine ratio in the soleus and tibialis anterior muscles from Sprague-Dawley rats treated with the selective PPARδ agonist GW610742 has been detected using *in vivo *^1^H NMR spectroscopy [17]. In addition, an increase in the concentrations of creatine and phosphocreatine were detected in the gastrocnemius of *ob*/*ob *mice following pharmacological activation of PPARδ [14].

Intriguingly, this increased oxidative capacity also manifested itself in increased amino acid metabolism, as indicated by the decrease in BCAAs in the cell culture media from PPARδ agonist-treated 3T3-L1 cells. PPARδ agonists have been linked to muscle atrophy [18], and one potential cause is the increased oxidation of amino acids, producing cachexia through increased protein turnover. Metabolomic profiling of obese versus lean humans has also recently indicated that BCAA concentrations are increased in obesity in the context of high fat consumption [19], which may be correlated with decreased PPARδ activity. BCAAs have also been causally implicated in the pathogenesis of insulin resistance [19,20], indicating that one possible mechanism by which PPARδ improves insulin resistance is by reducing the concentration of BCAAs.

In contrast to the PPARδ-mediated upregulation of oxidative pathways, PPARγ activation has previously been linked to an increase in glucose uptake and glycolysis in white adipose tissue [2,21]. A consistent observation from both the white adipose tissue and 3T3-L1 adipocytes exposed to the PPARγ agonist was a decrease in the concentration of glucose and other carbohydrate species. Transcriptomic analysis of the 3T3-L1 cells showed that the enzymes of glycolysis were increased in expression, but the labeling of lactate from 1-^13^C glucose was decreased compared with the control group. This was also associated with an increase in the concentrations of citrate and succinate and decreases in fumarate and malate. The 1-^13^C glucose labeling experiment and increased citrate concentration demonstrate that the increased glycolytic flux, and hence ability to metabolize extracellular glucose, is in fact associated with fatty acid synthesis. In addition, the microarray analysis of mRNA expression in 3T3-L1 adipocytes demonstrated a decrease in expression of isocitrate dehydrogenase, which in turn will stimulate the export of citrate out of mitochondria into the cytosol for fatty acid synthesis. Transcriptomic analysis also highlighted the significant upregulation in the expression of genes involved in calcium and calmodulin signaling within adipocytes treated with the PPARγ agonist GW347845. Calcium signaling increases GLUT4 translocation to the plasma membrane, increasing glucose transportation into adipocytes [2].

PPARγ activation also decreased flux through β-oxidation, as demonstrated by both decreased intracellular concentrations of carnitine and reduced labeling of TCA cycle intermediates and shorter chain fatty acids from U-^13^C palmitate. In addition, the expression of acyl-CoA thioesterase 7 and nudix-type motif 19 coenzyme A diphosphatase enzymes, which catalyze the hydrolysis of medium- and long-chain acyl-CoAs to FFA and CoA, and therefore prevent the β-oxidation of medium- and long-chain fatty acids once they are formed, was upregulated in the treated adipocytes. Transcription of nuclear receptor corepressor (Ncor1), a transcriptional repressor indicated in the downregulation of gluconeogenesis, oxidative and ketotic metabolism and lipolysis [22], was also increased in the adipocytes treated with the PPARγ agonist.

In contrast to the differences between fatty acid oxidation and synthesis associated with PPARδ and PPARγ stimulation, respectively, the changes induced in the desaturation of fats by the different agonists showed a high degree of similarity. Both PPARδ and PPARγ stimulation increased flux through stearoyl-CoA desaturase, a Δ-9 desaturase of saturated fatty acids under PPAR expressional control [23]. For PPARδ, the metabolism of U-^13^C palmitate in 3T3-L1 adipocytes demonstrated increased synthesis of palmitoleate from palmitate, while the normalized microarray data for stearoyl-CoA desaturase and fatty acid desaturase 3 show increased expression. PPARγ stimulation similarly upregulated the expression of stearoyl-CoA desaturase. The ω-3 and ω-6 essential fatty acid pathways were also upregulated by both PPARδ and PPARγ stimulation, as exemplified by total fatty acid content and transcriptional changes. The Δ 6-desaturase is integral to both pathways; the enzyme introduces the initial double bond into linoleate forming γ-linolenate in the ω-6 pathway and introduces the double bond into linolenate forming stearidonic acid. The Δ 6-desaturase gene is known to contain a peroxisome proliferator response element and is under PPAR transcriptional control [24] and may be the point of transcriptional control for both receptors within the essential fatty acid pathways.

The fundamental differences in fatty acid metabolism between the two agonists had a profound effect on the remodeling of triglycerides within adipose tissue. A decrease in the concentration of several TAGs was observed in the 3T3-L1 adipocytes following PPARδ activation. Within white adipose tissue several free fatty acids, such as palmitic acid, were increased in concentration despite their total concentration across lipid species within the tissue decreasing. These metabolic alterations are complicit with previously observed changes indicating that PPARδ activation in white adipose results in an increase in lipolysis in the tissue [25]. The results are corroborated by our observation, made using heteronuclear single quantum coherence (HSQC) NMR spectroscopy, that there was a decrease in the enrichment of glycerol in adipocytes incubated with 1-^13^C glucose and treated with PPARδ agonist when compared to control cells, indicating reduced synthesis of glycerol from glucose on activation of PPARδ. This, in part, could also explain the decrease in the concentration of TAGs due to reduced synthesis. Thus, PPARδ activation leads to a mobilization of lipid stores and concomitant decrease in the synthesis of the complex lipids such as TAGs required for fatty acid storage.

A distinct restructuring of the TAG pool also occurred as a consequence of PPARγ activation; the length and desaturation of fatty acids esterified to TAGs in cultured adipocytes was increased due to increased activities of fatty acid elongase and Δ-9 desaturase in white adipose tissue. PPARγ also directly regulates the *glycerol kinase *promoter and therefore promotes the esterification of fatty acids into TAGs [2]. Microarray analysis indicated that expression of genes encoding enzymes that catalyze the formation of phospholipids from lysophospholipids (lysophosphatidylcholine acyltransferase 3 acyltransferase), with a bias for incorporation of polyunsaturated fatty acid moieties, was increased in adipocytes treated with PPARγ [26]. In addition, the expression of mRNA encoding platelet activating factor acetylhydrolase 2 lipase, a lipase selective for phospholipids with short acyl chains at the sn-2 position, and angiopoietin-related protein 4, an inhibitor of lipoprotein lipase and therefore lipolysis, was increased in the PPARγ agonist-treated 3T3-L1 cells [27]. *Angiopoietin-related protein 4 *is a known target gene for PPARδ in muscle, and its apparent upregulation in adipose tissue by PPARγ identified in this study may give further insight into the tissue-specific targets of the PPAR isoforms [28]. Furthermore, the upregulation of the transcription of genes involved in calcium signaling as a consequence of PPARγ activation may play a role in defining the constituents of the complex lipid pool within the adipocytes. An increase in intracellular calcium stimulates the activity of fatty acid synthase, stimulates lipogenesis, inhibits basal lipolysis, and promotes TAG accumulation within murine and human adipocytes [2].

The concentrations of several metabolites in the polyol pathway were decreased following PPARδ activation, presumably attributable to increased glucose catabolism. On the other hand, PPARγ activation increased the production of glucitol and fructose, with sorbitol dehydrogenase being increased in expression in adipocytes following treatment. Given the role that the polyol pathway and aldose reductase have in the formation of toxic advanced glycation end-products and the resultant diabetic complications, such as neuropathy, nephropathy and retinopathy, a decrease in the activity of this pathway may prove a significant anti-diabetic effect of PPARδ activation [29].

One of the most striking differences between the two agonists *in vivo *was the mobilization of TAGs derived from C16:0, C18:0 and C18:1 fatty acids, representing in part the most highly synthesized fatty acids from glucose, in blood plasma by PPARδ activation and a reduction in the same TAGs by PPARγ activation. This would appear to be contrary to previous studies reporting PPARδ activation to be associated with a reduction of TAGs in blood plasma as a result of increased oxidation in skeletal muscle and white and brown adipose tissue [10,11,30]. However, it should be noted that different studies have used different agonists with differing relative doses and regimes, and so the effectiveness of short term lipid lowering in blood plasma may be variable. The detected increase in certain TAGs containing C16 and C18 saturated and monounsaturated fats in blood plasma, and a concomitant decrease in adipose tissue, following PPARδ activation most likely indicate an increase in mobilization of TAG stores as a result of increased oxidation of fats in skeletal muscle. However, as the mobilization of fat stores continues across time, presumably these TAGs in the blood plasma will decrease in concentration, reflecting the anti-atherogenic properties of PPARδ agonists.

## Conclusions

It has been shown that the anti-diabetic and anti-obesity effects of PPARδ activation are brought about, in part, by a decrease in fatty acid synthesis and fat storage within synthesized TAG depots and a concomitant mobilization of complex lipid fat stores. The mobilization of lipid energy stores is accompanied by upregulation of not only fatty acid oxidation but also carbohydrate and amino acid oxidative metabolism in white adipose, a tissue not traditionally thought of as being energetic and oxidative. This novel finding demonstrates PPARδ's ability to control global oxidation within adipose tissue. Essential to this process is the integration and co-ordination of the energy metabolism pathways, which PPARδ accomplishes by upregulating the transcription of a series of genes involved in glycolysis, the TCA cycle, the electron transport chain and fatty acyl β-oxidation. This is in marked contrast to PPARγ activation, where metabolic restructuring increases fatty acid synthesis in addition to the ultimate sequestration of fatty acids into triglycerides. Thus, while both agonists alleviate the effects of T2DM by potentially decreasing the lipid load on peripheral tissue and the induction of insulin resistance by lipotoxicity [31], stimulation of PPARδ may also reduce obesity, thus being a potent target for the treatment of the metabolic syndrome.

## Materials and methods

### *Ob*/*Ob *mouse study and tissue collection

All animal studies were performed within the relevant local legislation. Two-month-old male *ob*/*ob *mice (Jackson Labs, Bar Harbor, ME, USA) were fed standard laboratory chow *ad libitum *under controlled temperature, lighting and humidity. During the studies, body weight and food consumption (cage average) were recorded. The *ob*/*ob *mice were weight matched (mean weight 46 ± 1 g), assigned to three groups of eight and dosed orally daily with vehicle control, the PPARδ agonist GW610742 (30 mg/kg) or the PPARγ agonist GW347845 (5 mg/kg). Serum was collected via cardiac stick under isoflourane anesthesia at completion of the study on day 15. White adipose tissue (gonadal fat pad) was rapidly dissected (< 60 s post mortem), snap frozen in liquid nitrogen and stored at -80°C until extraction.

### 3T3-L cell culture and PPAR activation

3T3-L1 preadipocytes were grown in T75 flasks and maintained in DMEM (high glucose 4.5 g/l; Sigma-Aldrich, Gillingham, Dorset, UK) supplemented with 10% (v/v) new born calf serum (Sigma-Aldrich), 50 units/ml penicillin, and 50 μg/ml streptomycin (Sigma-Aldrich) in a humidified 5% CO_2 _incubator at 37°C. At 2 days post-confluence cells were induced to differentiate with DMEM supplemented with 10% (v/v) fetal bovine serum (FBS; Invitrogen, Paisley, Renfrewshire, UK), 1 μM dexamethasone (Sigma-Aldrich), 0.5 mM isobutylmethylxanthine (Sigma-Aldrich), 100 nM insulin (Sigma-Aldrich), 50 units/ml penicillin, and 50 μg/ml streptomycin. The cells were maintained in this media for 72 h as this was found to improve the reproducibility of differentiation between flasks. After 72 h the medium was replaced with DMEM supplemented with 10% FBS, 100 nM insulin, 50 units/ml penicillin, and 50 μg/ml streptomycin. The medium was subsequently changed for DMEM supplemented with 10% FBS, 50 units/ml penicillin, and 50 μg/ml streptomycin every 48 h [32].

At day 11 post-induction the medium on the cells was replaced with DMEM supplemented with 10% FBS, 100 nM insulin, 50 units/ml penicillin, and 50 μg/ml streptomycin containing DMSO (control; *n *= 6), the PPARδ selective agonist GW610742 (*n *= 6 at 100 nM and 1 μM) or the PPARγ selective agonist GW347845 (*n *= 6 at 10 nM and 100 nM) for 2 days prior to cell collection and metabolite extraction. These doses were based on the specific affinities of the compounds for their respective receptors.

Cells were collected by removing the medium and washing each T75 flask with 10 ml of phosphate-buffered saline. Cells were then washed with 1.5 ml trypsin-EDTA solution (5 BAEE units trypsin/ml, 1.8 μg EDTA/ml; Sigma-Aldrich) for 2 minutes at 37°C to remove the cells from the surface of the flask. DMEM (8.5 ml) supplemented with 10% (v/v) new born calf serum, 50 units/ml penicillin, and 50 μg/ml streptomycin was added to each flask. The DMEM containing the cells was transferred to a falcon tube and centrifuged at 200 g for 2 minutes to pellet the cells. The remaining medium was removed and the cells washed with physiological saline (0.9% NaCl) solution, and 2 ml of media was stored for further analysis.

### Tissue and 3T3-L1 metabolite extraction

Metabolites were extracted from white adipose tissue and 3T3-L1 cells using a modified Bligh and Dyer method [33]. Frozen white adipose tissue (approximately 100 mg for NMR and approximately 50 mg for GC-MS analysis) was pulverized with liquid nitrogen. Methanol-chloroform (2:1, 600 μl) was added to the white adipose tissue, serum (50 μl), and to 5 mg cell pellets (3T3-L1 cells) and the samples were sonicated for 15 minutes. Chloroform-water (1:1) was then added (200 μl of each). Samples were centrifuged (16,100 g, 20 minutes) and the organic and aqueous phases were separated and stored at -80°C until analysis. Of these fractions, 100 μl of the organic phase was used for LC-MS, and the remaining organic phase was used for GC-MS. Prior to analysis the organic fractions were dried in a fume hood. For the aqueous phase, 100 μl of the aqueous phase was taken for GC-MS analysis, and the remaining aqueous phase sample was used for ^1^H NMR spectroscopy.

### ^1^H-NMR spectroscopy

Dried extracts were dissolved in 600 μl of D_2_O and buffered in 0.24 M sodium phosphate (pH 7.4) containing 1 mM TSP (sodium-3-(trimethylsilyl)-2,2,3,3-tetradeuteriopropionate; Cambridge Isotope Laboratories, Andover, MA, USA) and 0.02% sodium azide. Samples were analyzed using a DRX Avance II+ spectrometer interfaced to a 5-mm TXI ATMA probe (Bruker BioSpin GmbH, Rheinstetten, Germany) at a proton frequency of 500.13 MHz. A presaturation pulse sequence for water suppression based on a one-dimensional nuclear Overhauser effect spectroscopy pulse sequence was used to saturate the residual water proton signal (relaxation delay = 2 s, t_1 = _4 μs, mixing time = 50 ms). We collected 128 and 256 transients for white adipose tissue extracts and 3T3-L1 cell extracts, respectively, into 64 K data points over a spectral width of 8,000 Hz at 300 K. NMR spectra were processed in ACD 1D NMR Manager (Advanced Chemistry Development Inc., Toronto, Canada). The NMR spectra were integrated using 0.04 ppm integral regions between 0.2 and 9.56 ppm (excluding water resonance between 4.20 and 5.08 ppm). Spectra were normalized to total integrated area to account for differences in concentration between samples and assigned by comparison with previous literature and Chenomx NMR suite 5.0 libraries.

### GC-MS analysis

Dried aqueous phase samples were derivatized using methoxyamine hydrochloride solution (20 mg/ml in pyridine; Sigma-Aldrich) and 30 μl of *N*-methyl-*N*-trimethylsilyltrifluoroacetamide (Macherey-Nagel, Duran, Germany) using the method described by Gullberg *et al. *[34].

Acid-catalyzed esterification was used to derivatize the organic phase samples. Chloroform-methanol (1:1, 0.25 ml) and BF_3_-methanol (10%, 0.125 ml) was added to the organic phase and incubated at 90°C for 90 minutes. Water (0.15 ml; ultrapurified to resistivity 18.2 MΩ cm) and hexane (0.3 ml) were added and the samples vortex mixed for 1 minute and left to form a bilayer. The aqueous phase was discarded and the organic layer evaporated to dryness prior to reconstitution in analytical grade hexane (200 μl) before GC-MS analysis. All GC-MS analyses were made using a Trace GC Ultra coupled to a Trace DSQ II mass spectrometer (Thermo Scientific, Hemel Hempstead, UK). Derivatized aqueous samples were injected splitless onto a 30 m × 0.25 mm 5% phenylpolysilphenylene-siloxane column with a 0.25 μm ZB-5 ms stationary phase (Phenomenex, Macclesfield, Cheshire, UK) as described in Roberts *et al. *[32]. Full-scan spectra were collected using three scans/s over a range of 50 to 650 *m*/*z*.

The derivatized organic samples were injected with a split ratio of 60 for white adipose tissue and 8 for 3T3-L1 cells onto a 30 m × 0.25 mm 70% cyanopropyl polysilphenylene-siloxane 0.25 μm TR-FAME stationary phase column (Thermo Scientific) as described above and by Roberts *et al. *[32].

GC-MS chromatograms were processed using Xcaliber (version 2.0; Thermo Scientific). Each individual peak was integrated and then normalized. Overlapping peaks were separated using traces of single ions. Peak assignment was based on mass fragmentation patterns matched to the National Institute of Standards and Technology (USA) library and to previously reported literature. Identification of metabolites from organic phase GC-MS analysis was supported by comparison with a FAME standard mix (Supelco 37 Component FAME Mix; Sigma-Aldrich).

### Ultra performance LC-MS analysis

Chromatography was performed using an ACQUITY UPLC^® ^system (Waters Corporation, Centennial Park, Elstree, Hertfordshire) equipped with an Acquity UPLC 1.7 μm Bridged Ethyl Hybrid (BEH) C8 column (2.1 × 100 mm Waters), which was kept at 65°C and coupled to a Micromass QTof-Ultima™ with a Z-spray™ electrospray source as described by Roberts *et al. *[32]. The binary solvent system used was solvent A comprising HPLC grade water (Sigma-Aldrich), 1% 1 M ammonium acetate (NH_4_Ac; Sigma-Aldrich) and 0.1% formic acid (Sigma-Aldrich) and solvent B comprising analytical grade acetonitrile (chromosolv, Sigma-Aldrich)/isopropanol (Fisher Scientific, Loughborough, Leicestershire, UK) 5:2, 1% 1 M NH_4_Ac, and 0.1% formic acid [35]. Mass spectrometric data were collected in full scan mode from 100 to 1,350 *m*/*z *for adipose and 100 to 1,500 *m*/*z *for 3T3-L1 cells from 0 to 14 minutes with a scan duration of 0.5 s and an interscan delay of 0.1 s.

For tissue extracts, the column mobile phase was held at 85% solvent B for 0.5 minutes followed by an increase from 85 to 100% solvent B over 0.5 to 8 minutes. The mobile phase was then held at 100% solvent B for 4 minutes. Between 12 and 12.25 minutes the mobile phase was returned to 85% solvent B held for 1.75 minutes to re-equilibrate the column. For serum extracts, the column mobile phase was held at 70% solvent B for 0.5 minutes followed by an increase from 70 to 100% solvent B over 0.5 to 6.5 minutes. The mobile phase was then held at 100% solvent B for 3.5 minutes. Between 10 and 10.25 minutes the mobile phase was returned to 70% solvent B held for 3.75 minutes to re-equilibrate the column. For 3T3-L1 cell organic phase metabolites, the column mobile phase was held at 50% solvent B for 0.5 minutes followed by an increase from 50 to 100% solvent B over 0.5 to 6.5 minutes. The mobile phase was then held at 100% solvent B for 3.5 minutes. Between 10 and 10.25 minutes the mobile phase was returned to 50% solvent B held for 3.75 minutes to re-equilibrate the column. The total ultra performance liquid chromatography (UPLC) cycle was 14 minutes and the eluent flow rate was 600 μl/minute for both methods.

Tandem mass spectrometry (MS/MS) was used for the identification of selected lipids. Data were processed using Micromass Markerlynx Applications Manager (Waters Corporation).

### DI-MS analysis

Mass spectrometric analysis was also performed using a Thermo Finnigan LTQ equipped with a Finnigan Surveyor pump and Finnigan Micro AS Autosampler Thermo Finnigan, Hemel Hempstead, Hertfordshire, UK.

The 3T3-L1 organic phase samples for DI-MS were reconstituted in 500 μl methanol:tetrahydrofuran (2:1, v/v). Samples were analyzed in triplicate using both positive and negative mode. The scan range was set at 100 to 1,100 *m*/*z *in profile for both positive and negative mode. DI-MS chromatograms were processed using Xcaliber (version 2.0; Thermo Electron). The mass data were summed from the chromatogram for the period of sample injection and the exact masses were exported; the data points were summed between M and M+1, normalized to total metabolite concentration and integrated. Tandem mass spectrometry data were collected for identification purposes.

### ^13^C-glucose substrate labeling study

At 2 days post-differentiation medium was removed from the T75 flasks and replaced with DMEM (10% (v/v) fetal bovine serum, 50 units/ml penicillin, and 50 μg/ml streptomycin) and either 4.5 g/l unlabeled glucose with DMSO control (*n *= 6), 1 μM GW610742 (*n *= 7) or 1 μM GW347845 (*n *= 7), or 4.5 g/l 1-^13^C-glucose with DMSO control (*n *= 7), 1 μM GW610742 (*n *= 7) or 1 μM GW347845 (*n *= 7). After 2 days cells were collected and metabolites extracted as previously described.

### ^13^C-palmitate substrate labeling study

Palmitate was solubilized using a dialyzed albumin solution. At 2 days post-differentiation medium was removed from the T75 flasks and replaced with DMEM (serum free, 50 units/ml penicillin, and 50 μg/ml streptomycin) and either 70 μM unlabeled palmitate with DMSO control (*n *= 6), 1 μM GW610742 (*n *= 7) or 1 μM GW347845 (*n *= 7), or 70 μM U-^13^C labeled palmitate with DMSO control (*n *= 6), 1 μM GW610742 (*n *= 7) or 1 μM GW347845 (*n *= 7). After 2 days cells were collected and metabolites extracted as previously described.

### ^13^C-Heteronuclear single quantum coherence NMR

Dried organic phase extracts were dissolved in 600 μl of deuterated chloroform. Samples were analyzed using a DRX Avance II+ spectrometer interfaced to a 5-mm TXI ATMA probe. Analysis was performed using two-dimensional H-1/X correlation via double inept transfer with sensitivity improvement. Spectral widths of 10.00 ppm and 160 ppm were used in the F2 (1H) and F1 (13C) dimensions, respectively, with an offset of 75.00 ppm. Spectra were acquired using 96 scans with a relaxation delay of 1.0 s. Datasets were zero-filled and multiplied by sine bell squared functions prior to Fourier transformation.

### ^13^C-labeled substrate GC-MS analysis

Analysis of organic and aqueous phases was carried out as previously described above. Enrichment of metabolites was identified by calculating isotope ratios of the M and M+1 ions for the parent ion of the fragmentation pattern in the case of ^13^C-glucose metabolism analysis and TCA cycle intermediates originating from ^13^C-palmitate oxidation. For fatty acid synthesis and desaturation products from ^13^C-labeled palmitate an ion ratio of M+16/M was used, and for fatty acids originating from oxidation of ^13^C-labeled palmitate an ion ratio of M+n/M was used, where *n *= the carbon chain length of the fatty acid. Statistical analysis was performed using a univariate Student *t*-test.

### Multivariate analysis

Multivariate data analysis was performed using SIMCA-P^+ ^11.0 (Umetrics AB, Umeå, Sweden). NMR, DI-MS and UPLC-MS data sets were mean-centered and Pareto-scaled prior to analysis. GC-MS data sets were scaled to unit variance (UV) as only manually fitted peaks were analyzed. Data sets were analyzed using principal components analysis and PLS-DA. Metabolite changes responsible for clustering or regression trends within the pattern recognition models were identified by interrogating the corresponding loadings plot. Metabolites identified in the variable importance in projections/coefficients plots were deemed to have changed globally if they contributed to separation in the models with a confidence limit of 95%.

### Respirometric analysis of PPARδ and PPARγ agonist-treated 3T3-L1 cells

Cells were grown, treated with either vehicle control, the PPARδ agonist or the PPARγ agonist for 2 days prior to collection into respiration medium (100 mM KCl, 50 mM MOPS (3-(N-morpholino)propanesulfonic acid), 1.0 mM KH_2_PO_4_, 1.0 mg/ml defatted bovine serum albumin, pH 7.4). Respiratory rates of *in situ *permeabilized 3T3-L1 cells were measured using a Clark-type oxygen electrode (Strathkelvin Instruments Ltd, Glasgow, UK) [36]. Respiration rates were recorded and quantified using 782 Oxygen System v3.0 software (Strathkelvin Instruments). Oxygen concentrations were measured continuously in 0.5 ml respiration medium containing 250,000 cells in a respiration chamber maintained at 37°C for 40 minutes. The cells were initially permeabilized with the addition of Digitonin (25 μg/ml), before malate (5 mM) plus palmitoyl-carnitine (0.04 mM) were added as respiratory substrates to measure the fatty acid oxidation rates. Respiration was stimulated by the addition of a saturating concentration of ADP (2 mM) plus MgCl_2 _(0.6 mM) and subsequently measured. Antimycin (5 μM) was then added to inhibit complex III of the electron transport chain, and respiration ceased. Complex IV respiration was stimulated by addition of the artificial substrates TMPD (N, N, N', N'-tetramethyl-p-phenylenediamine dihydrochloride, 0.5 mM) and ascorbate (2 mM). Finally, respiration was terminated by addition of the complex IV inhibitor sodium azide (3 mM).

### Microarray analysis of PPARδ agonist-treated 3T3-L1 cells

3T3-L1 adipocytes were cultured and then treated with either the PPARδ agonist, the PPARγ agonist or vehicle control as described above (*n *= 6 independent individual hybridizations for each treatment group). RNA was extracted using RNeasy (Qiagen GmbH, Hilden, Germany). Approximately 5 mg of cells was used per sample for RNA isolation. Procedures were carried out according to the manufacturer's instructions. Extracted RNA was quantified and its purity assessed using a Nanodrop ND-1000 Spectrometer (Nanodrop Technologies Inc., Wilmington, NC, USA) to measure the absorbance at 260 nm and the A_260_/A_280 _ratio, respectively. Illumina Infinium Gene Expression BeadArrays (Illumina Inc., San Diego, CA, USA) were used to perform transcriptomics. A mouse WG6 array platform was used with 45,281 probes. Analysis was performed with R/BioConductor version 2.5. The R package lumi [37] was used with the detection *P*-value threshold set to 0.01. Probes were required to be successfully detected (*P*-value < 0.01 in Lumi) in at least one sample to pass the selection. The data were transformed using variance stabilization [38] and then normalized using quantile normalization. Gene expression was compared using the R package limma [39] with a 95% confidence interval. The selected and normalized data were then analyzed using Simca-P+. The 6% of transcripts most responsible for separation in the multivariate models were then examined (3% most increased and 3% most decreased in treated cells as identified in the multivariate models). The microarray data have been deposited with the Gene Expression Omnibus and have the accession number [GSE26207].

The Reactome Skypainter tool was used to determine which pathways were statistically significant in terms of key perturbations [13]. From a given set of genes participating in a pathway, the total genes for *Mus musculus *and the submitted genes (genes increased in PPARδ- or PPARγ-activated cells) of which N genes participate in a pathway, the probability of observing at least N genes from a pathway if that pathway is not overrepresented in the submitted list of genes is calculated using Fisher's exact test. A *P*-value smaller than the significance level suggests the pathway is significantly represented.

### Univariate statistical analysis methodology

Univariate analysis was performed using an unpaired Student's *t*-test with a significance level set to *P *< 0.05. An F-test was also utilized to compare the variance of two distributions. All univariate analysis was conducted in GraphPad Prism (version 4).

## Abbreviations

BCAA: branched chain amino acid; CoA: coenzyme A; DI: direct infusion; DMEM: Dulbecco's modified Eagles media; FBS: fetal bovine serum; GC: gas chromatography; HSQC: heteronuclear single quantum coherence; LC: liquid chromatography; PC: phosphatidylcholine; MS: mass spectrometry; NMR: nuclear magnetic resonance; PLS-DA: partial least squares-discriminant analysis; PPAR: peroxisome proliferator-activated receptor; T2DM: type 2 diabetes mellitus; TAG: triacylglycerol; TCA: tricarboxylic acid; UPLC: ultra performance liquid chromatography.

## Competing interests

The authors declare that they have no competing interests.

## Authors' contributions

LDR was responsible for document preparation, tissue culture, metabolomic, oxygen consumption, flux and statistical analysis. AJM and DM performed the oxygen consumption analysis. TA performed the metabolomic analysis of cell culture media. AWN was responsible for scientific discussion and guidance. JLG was responsible for document preparation, metabolomic analysis of cell culture media, scientific discussion and guidance. All authors have read and approved the manuscript for publication.

## Supplementary Material

Additional file 1 **Figure S1 - M+1/M isotope ratio ^13^C enrichment of lactate, succinate, glutamate and arachidate**. **(a-c) **M+1/M isotope ratio ^13^C enrichment of (a) lactate, (b) succinate and (c) glutamate analyzed by GC-MS of the aqueous fraction from control and PPARγ agonist-treated 3T3-L1 cells incubated with 1-^13^C-glucose. **(d) **M+1/M isotope ratio ^13^C enrichment of arachidate analyzed by GC-MS of the organic fraction from control and PPARγ agonist-treated 3T3-L1 cells incubated with 1-^13^C-glucose. *P < 0.05. The metabolites have been mapped to the glycolysis, TCA cycle and fatty acid synthesis metabolic pathways. An upward pointing arrows indicates a metabolite increased in ^13^C enrichment by PPARγ activation, and a downward pointing arrows indicates a metabolite decreased in ^13^C enrichment by PPARγ activation. Parent ions were used to calculate ion ratio. Click here for file

Additional file 2 **Figure S2 - the average integrated area of the two-dimensional HSQC-NMR organic fraction glycerol peak**. **(a) **A graph of the average integrated area of the two-dimensional HSQC-NMR organic fraction glycerol peak (^13^C chemical shift 62.04) from control and 1 μM PPARδ agonist-treated 3T3-L1 adipocytes incubated in 1-^13^C glucose. **(b) **A graph of the average integrated area of the two-dimensional HSQC-NMR organic fraction glycerol peak (^13^C chemical shift 62.17) from control and 1 μM PPARδ agonist-treated 3T3-L1 adipocytes incubated in 1-^13^C glucose. **(c) **A graph of the average integrated area of the two-dimensional HSQC-NMR organic fraction esterified glycerol peak from control and 1 μM PPARδ agonist-treated 3T3-L1 adipocytes incubated in 1-^13^C glucose. *P < 0.05, ***P < 0.005. Parent ions were used to calculate ion ratio. Click here for file

Additional file 3 **Figure S3 - M+1/M isotope ratios**. **(a, b) **The M+1/M isotope ratio ^13^C enrichment of (a) glutamate and (b) isocitrate analyzed by GC-MS of the aqueous fraction from control and PPARγ agonist-treated 3T3-L1 cells incubated with ^13^C-U-palmitate. **(c-e) **Graphs showing the isotope ratio ^13^C enrichment of myristate (c), arachidate (d) and palmitate (e) analyzed by GC-MS of the organic fraction from control and PPARγ agonist-treated 3T3-L1 cells incubated with ^13^C-U-palmitate. The metabolites have been mapped to the TCA cycle and fatty acid β-oxidation/synthesis metabolic pathways. Red indicates a metabolite increased in ^13^C enrichment by PPARγ activation. Blue indicates a metabolite decreased in ^13^C enrichment by PPARγ activation. *P < 0.05, **P < 0.01,***P < 0.005. Parent ions were used to calculate ion ratio. Click here for file

Additional file 4 **Figure S4 - **t**he effect of PPARγ activation on the integration of the energy metabolism pathways of 3T3-L1 adipocytes**. A diagram showing the effect of PPARγ activation on the integration of the energy metabolism pathways of 3T3-L1 adipocytes based on the combination of results from the metabolomic, transcriptomic and stable isotope labeling studies. Red indicates an increase in concentration or expression in cells treated with the PPARγ selective agonist GW347845. Blue indicates a decrease in concentration in cells treated with the PPARγ selective agonist GW347845. Click here for file
